# Syndrome of inappropriate antidiuretic hormone secretion following extensively drug-resistant *Klebsiella pneumoniae* associated with complicated urinary tract infection: a case report

**DOI:** 10.3389/fmed.2025.1574251

**Published:** 2025-06-25

**Authors:** Junqing Li, Zhuo Yang, Kai Wang, Bingbing Zha, Yong Du, Jindong Shi, Zhijun Jie, Heyuan Ding

**Affiliations:** ^1^Department of Respiratory and Critical Care Medicine, Shanghai Fifth People’s Hospital, Fudan University, Shanghai, China; ^2^Center of Community-Based Health Research, Fudan University, Shanghai, China; ^3^Department of Endocrinology, Shanghai Fifth People’s Hospital, Fudan University, Shanghai, China; ^4^Department of Endocrinology, Luxi County People’s Hospital, Yunnan, China; ^5^Department of Endocrinology, Shanghai Xuhui Central Hospital, Fudan University, Shanghai, China

**Keywords:** SIADH, hyponatremia, extensively drug-resistant, *Klebsiella pneumoniae*, urinary tract infection

## Abstract

Hyponatremia is strongly associated with syndrome of inappropriate antidiuretic hormone secretion (SIADH). We present an 82-year-old male with refractory hyponatremia unresponsive to the discontinuation of hydrochlorothiazide and comprehensive treatment (fluid restriction, sodium supplementation, and tolvaptan). Two consecutive urine cultures identified extensively drug-resistant *Klebsiella pneumoniae* (XDRKP). After tigecycline treatment, serum sodium returned to normal and remained stable during one-year follow-up. This case suggests a close relationship between XDRKP infection and SIADH, highlighting the need to evaluate drug-resistant pathogens in patients with refractory hyponatremia and the importance of antimicrobial therapy in the management of electrolyte disorders secondary to drug-resistant urinary tract infections.

## Introduction

Hyponatremia is the most common electrolyte disturbance in clinical practice, occurring in 15 to 30% of hospitalized patients (1). Hyponatremia is associated with many adverse outcomes, such as osteoporosis, fractures, falls, heart failure, increased mortality and morbidity, prolonged hospital stays (2–6). The syndrome of inappropriate antidiuretic hormone secretion (SIADH) is the leading cause of hyponatremia in both hospitalized and ambulatory patients (7–9).

## Case presentation

An 82-year-old man presented to the emergency department with one-month history of productive cough and fever. Physical examination showed poor mental state, heavy breath sounds, and no peripheral edema. The patient had hypertension and was taking irbesartan hydrochlorothiazide (150 mg/12.5 mg qd) before admission. The patient also had prostatic hyperplasia and dysuria. Laboratory test showed that the serum sodium was 117 mmol/L on the admission day. Lung CT scan showed mild infection in the upper lobes of both lungs. Ultrasound of the urinary tract showed no significant obstructive disease. According to the patient’s condition, irbesartan hydrochlorothiazide was discontinued and symptomatic treatments such as sodium supplement, tolvaptan (15 mg, bid), imipenem/cilastatin (1.0 g, q12h), and irbesartan (150 mg, qd) were given. The patient was also instructed to limit water intake.

**Figure 1:**
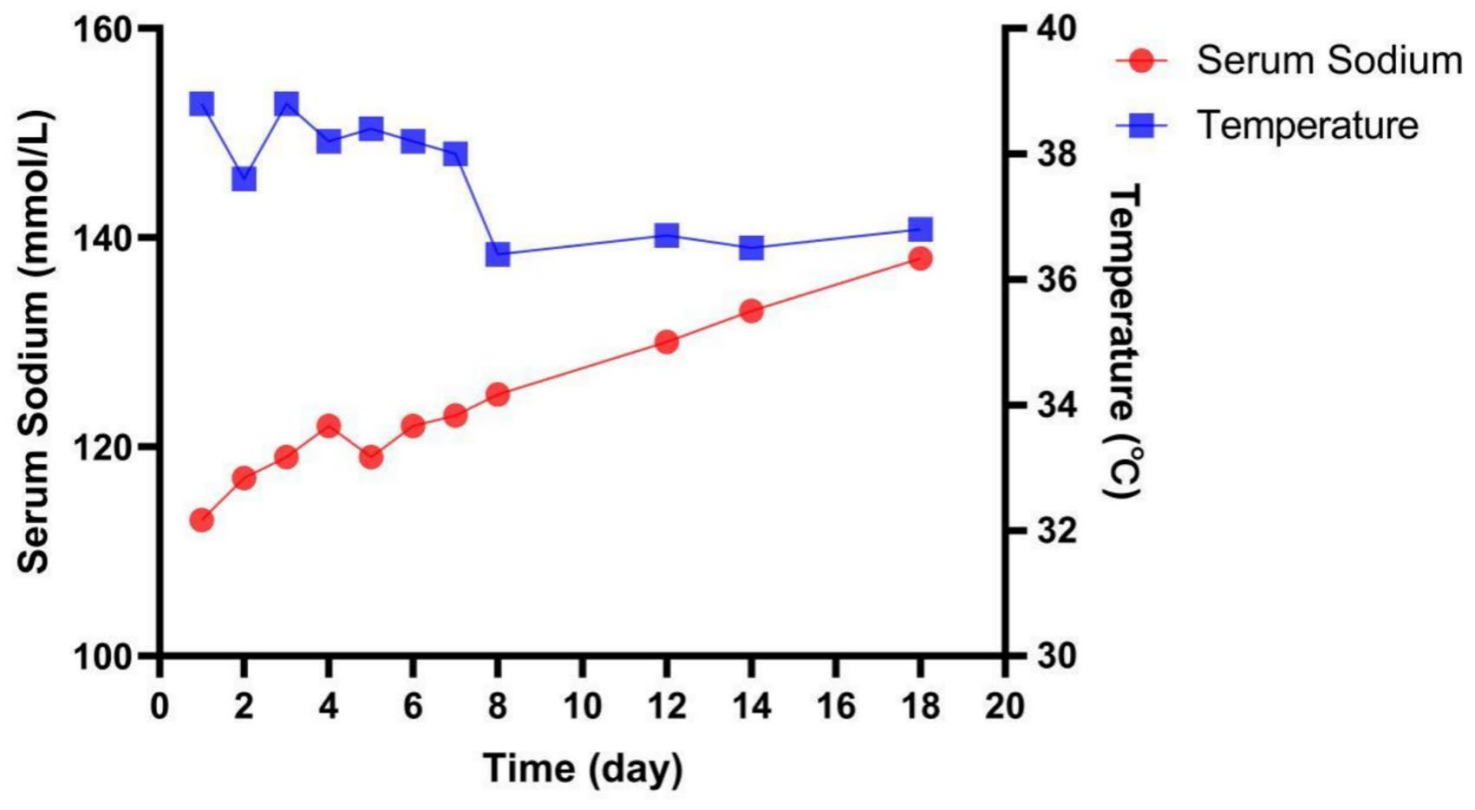
Temperature and serum sodium concentration over time. Serum sodium level was 117 mmol/L on the admission day. Initially sodium supplementation and tolvaptan was attempted. Given the lack of response, tigecycline (50 mg q12h) was given to treat the infection. Serum sodium began to rise gradually, and finally back to normal.

After treatments, pulmonary infection was significantly improved, but hyponatremia and fever were not significantly improved. Meanwhile, the patient developed urinary retention and urinary catheterization was performed. Routine urine examination showed complicated urinary tract infection, and two consecutive urine cultures found extensively drug-resistant *Klebsiella pneumoniae* (XDRKP). Sputum cultures did not reveal pathogenic bacteria. Thus, imipenem/cilastatin was discontinued and tigecycline (50 mg q12h) was given to for anti-infection on the seventh day of admission. The patient’s serum sodium level was 117 mmol/L on admission and ranged from 117 to 122 mmol/L before tigecycline was administered. The patient’s body temperature returned to normal and serum sodium began to rise on the eighth day of admission. The patient’s serum sodium completely returned to normal on the eighteenth day (Figure 1). His laboratory data were summarized in Table 1. The diagnosis of SIADH should be based on clinical manifestations, laboratory tests, imaging and medical history, and other causes of hyponatremia should be excluded. One year after discharge, the patient’s serum sodium level was 143 mmol/L.

**Table 1:** Laboratory values, including initial presentation, and hospital course, including serum and urine electrolytes, as well as serum and urine osmolality.

Lab element (normal value)	Hospital day						
	1	2	6	10	12	14	18
Serum Na, mmol/L (135–145 mmol/L)	117	119	122	121	130	136	141
Serum K, mmol/L (3.5–5.5 mmol/L)	4.2	4.3	4.1	3.5	3.6	4.5	3.6
Serum Cl, mmol/L (96–108 mmol/L)	86	86	86	89	93	101	104
Serum UN, mmol/L (2.78–8.07 mmol/L)	6.5	5.7	5		3.5	7.5	3.6
Serum Crea, μmol/L (59–104 μmol/L)	88	76	81		75	92	69
Serum OSM, mOsm/kg (275–295 mOsm/kg)	246.6	251.6	256.7	254.4	271.7	285.3	293.6
Urine Na, mEq/L (20 mEq/L)		123		121			
Urine K, mEq/L (20 mEq/L)		17.3		16.3			
Urine Crea, mg/dL (39–259 mg/dL)		4,464					
Urine Osm, mOsm/kg (300–1,000 mOsm/kg)		561.2		549.2			
TSH, uIU/mL (0.270–4.20 mIU/L)		1.47					
Cortisol, pmol/l (138–480 pmoL/L)		403					

Na, sodium; K, potassium; Cl, Chlorine; UN, urea nitrogen; Crea, creatinine; OSM, osmolality; TSH, thyrotropin.

## Discussion

SIADH is a common cause of hyponatremia, which can be secondary to malignancy, central nervous system disorders, medications, pulmonary diseases, and inflammatory diseases (10–13). In this case, although the patient had used thiazides before admission, irbesartan hydrochlorothiazide had been discontinued on admission. The treatments such as sodium supplementation, fluid restriction, and anti-infection were given. After comprehensive treatment, the patient’s pneumonia and urinary retention had improved, but the serum sodium level remained low. Ultrasound of the urinary tract showed no significant obstructive disease. Other tests revealed no evidence of adrenal insufficiency, hypothyroidism, malignancy, or central nervous system disease. Serum sodium began to rise after tigecycline treatment, suggesting a close relationship between hyponatremia and XDRKP infection. One year later, the examination of blood sodium was normal. These evidences indicated that XDRKP infection was the cause of SIADH.

In 2013, Babar SM reported that a 68-year-old Caucasian woman experienced two episodes of SIADH during ciprofloxacin treatment for a urinary tract infection (14). In that case, the ciprofloxacin was discontinued on admission, and her sodium levels rose. Therefore, the cause of SIADH in the case was considered to be related to ciprofloxacin treatment and not to the urinary tract infection. Another case was reported by Ropero-Luis G in 2023, which a 54-year-old man presented with symptoms of dysuria and cloudy urine, as well as a history of passing stool in urine and recurrent urinary tract infections over the past 3 months, with a final diagnosis of SIADH with bladder fistula due to chronic diverticulitis perforation (15).

Urinary tract infection is a common cause of SIADH. At present, the mechanism between urinary tract infection and hyponatremia has not been fully elucidated (7). Lipopolysaccharide (LPS) and inflammatory mediators (such as IL-1β, IL-6, and TNF-*α*) contribute to the pathogenesis of hyponatremia. LPS and inflammatory mediators stimulate AVP neurons in the supraoptic nucleus (SON) and paraventricular nucleus (PVN) of the hypothalamus, resulting in increased AVP secretion (16–19). AVP acts on V2 receptors in the renal collecting duct to activate the cAMP-PKA pathway, enabling the aquaporin AQP2 to embed into the luminal membrane. This results in increased water reabsorption, urinary concentration (increased urinary osmolal pressure), and inhibition of the renin-angiotensin-aldosterone system (RAAS), which leads to the development of hyponatremia (20). *Klebsiella pneumoniae* (KP) is one of the most clinically relevant species responsible for community-acquired and nosocomial infections in immunocompromised individuals, including pneumonias, urinary tract infections, bacteremia, and liver abscesses (21). Carbapenems are important antibacterial drugs for the treatment of such infections. However, in recent years, the isolation rate of carbapenem-resistant *Klebsiella pneumoniae* (CRKP) has gradually increased. XDRKP was defined as *Klebsiella pneumoniae* that is only sensitive to one or two classes of antibacterial drugs.

SIADH is a biochemical and clinical syndrome of euvolemic hyponatremia, occurring when the antidiuretic effect of arginine vasopressin is enhanced (22). Therefore, volume status can assist in the diagnosis of hyponatremia caused by different causes. Measuring serum osmolality is very useful when plasma sodium is below 135 mmol/L for no apparent reason. SIADH should be suspected in patient with hypoosmotic hyponatremia (low plasma osmolality) and urine osmolality >100 mOsm/kg (23).

The diagnosis of SIADH requires the exclusion of other possible causes of hyponatremia and increased urinary sodium, and must meet some criteria. At present, the criteria described in the first cases published by Schwartz and Bartter and re-issued by Schwartz et al. is still used (13). Management of SIADH begins with a good clinical history and physical examination, as well as laboratory tests, which are essential to discover the details and determine the cause. Once the diagnosis of SIADH, appropriate treatments can be determined based on the cause.

## Conclusion

Although the relationship between XDRKP and SIADH remains to be investigated, it is necessary to master the diagnosis and treatment of hyponatremia in clinical practice.

## Data Availability

The original contributions presented in the study are included in the article/supplementary material, further inquiries can be directed to the corresponding authors.
